# Case-only approach applied in environmental epidemiology: 2 examples of interaction effect using the US National Health and Nutrition Examination Survey (NHANES) datasets

**DOI:** 10.1186/s12874-022-01706-6

**Published:** 2022-09-29

**Authors:** Jinyoung Moon, Hwan-Cheol Kim

**Affiliations:** 1 Department of Environmental Health Science, Graduate School of Public Health, Seoul National University, Gwanak-ro 1, Gwanak-gu, Seoul, 08826 Republic of Korea; 2 Department of Occupational and Environmental Medicine, Inha University Hospital, Inhang-ro 27, Jung-gu, Incheon, 22332 Republic of Korea; 3 Department of Social and Preventive Medicine, College of Medicine, Inha University, Inha-ro 100, Michuhol-gu, Incheon, 22212 Republic of Korea

**Keywords:** Case-only approach, Environmental epidemiology, Interaction effect, Independence assumption, National Health and Nutritional Examination Survey, Susceptibility factor, Environmental exposure

## Abstract

**Introduction:**

By substituting the general ‘susceptibility factor’ concept for the conventional ‘gene’ concept in the case-only approach for gene-environment interaction, the case-only approach can also be used in environmental epidemiology. Under the independence between the susceptibility factor and environmental exposure, the case-only approach can provide a more precise estimate of an interaction effect.

**Methods:**

Two analysis examples of the case-only approach in environmental epidemiology are provided using the 2015–2016 and 2017–2018 US National Health and Nutritional Examination Survey (NHANES): (i) the negative interaction effect between blood chromium level and glycohemoglobin level on albuminuria and (ii) the positive interaction effect between blood cobalt level and old age on albuminuria. The second part of the methods (theoretical backgrounds) summarized the logic and equations provided in previous studies about the case-only approach.

**Results:**

(i) When a 1 μg/L difference of both blood chromium level (mcg/L) and a 1% difference in blood glycohemoglobin level coincide, the multiplicative interaction contrast ratio (ICR_c/nc_) was 0.72 (95% CI 0.35–1.60), with no statistical significance. However, when only the cases were analyzed, the case-only ICR (ICR_CO_) was 0.59 (95% CI 0.28–0.95), with a statistical significance (a negative interaction effect). (ii) When a 1 μg/L difference of both blood cobalt levels and a 1-year difference in age coincide, the multiplicative interaction contrast ratio (ICR_c/nc_) was 1.13 (95% CI 0.99–1.37), with no statistical significance. However, when only the cases were analyzed, the case-only ICR (ICR_CO_) was 1.21 (95% CI 1.06–1.51), with a statistical significance (a positive interaction effect).

**Discussion:**

The discussion suggested the theoretical background and previous literature about the possible protective interaction effect between blood chromium levels and blood glycohemoglobin levels on the incidence of albuminuria and the possible aggravating interaction effect between blood cobalt levels and increasing ages on the incidence of albuminuria. If the independence assumption between a susceptibility factor and environmental exposure in a study with cases and non-cases is kept, the case-only approach can provide a more precise interaction effect estimate than conventional approaches with both cases and non-cases.

**Supplementary Information:**

The online version contains supplementary material available at 10.1186/s12874-022-01706-6.

## Introduction

The estimation of an interaction effect has often been conducted in cohort or case-control studies using information from both cases and controls [1–4]. However, a case-only approach can be a valid alternative and even may have advantages under certain circumstances over conventional approaches that use information from both cases and controls.

The case-only approach is used to calculate the interaction effect estimate. This unique approach is mainly used in gene-environmental and gene-gene interaction studies in genetic epidemiology [5–8]. However, if the ‘gene’ concept in the gene-environmental interaction could indicate a type of ‘susceptibility factor,’ the term ‘gene-environment interaction’ in genetic epidemiology can be replaced with the ‘susceptibility factor-environmental exposure interaction’ in environmental epidemiology.

The case-only approach can provide 2 benefits over a study with cases and non-cases or conventional cohort/case-control studies to estimate the interaction effect between a susceptibility factor and an environmental exposure [5, 7–13]. The first is that a more precise interaction effect estimate can be calculated. The second is that this approach can estimate the interaction effect when appropriate controls are unavailable. However, this case-only approach requires an important condition between the susceptibility factor and the environmental exposure studied: independence [5, 14]. If this independent assumption between a susceptibility factor and an environmental exposure is not fulfilled, the case-only interaction estimate might be biased severely from the interaction effect estimate acquired from a study with cases and non-cases.

This study will summarize all logic, definitions, and equations about the case-only approach through various study types, including case-only studies and a study with cases and non-cases, including case-control and cohort studies. In addition, this study will deal with important assumptions and the relationship among these assumptions, which are required for the reliable estimation of the interaction effect in the case-only approach. Possible corrective strategies for the violation of the independence assumption will also be dealt with. Finally, 2 analysis examples of the case-only approach will be illustrated using the US NHANES dataset. This study can clarify the logic and equations of the case-only approach and contribute to applying the case-only approach of genetic epidemiology to environmental epidemiology.

## Methods: application for real data – 2 examples

In this study, 2 analysis examples using the US National Health and Nutritional Examination Survey (NHANES) data will be provided (https://www.cdc.gov/nchs/nhanes/index.htm). The case-only approach applied in environmental epidemiology will be explained using this dataset.

### The preventive (negative) interaction effect between blood chromium level and glycohemoglobin level on albuminuria (micro and macro)

The laboratory data of NHANES 2015–2016 and NHANES 2017–2018 datasets were used. The blood chromium levels (mcg/L) were used as the environmental exposure variable, and the glycohemoglobin levels (%) were used as the susceptibility factor variable. The albumin creatinine ratio (mg/g) was the outcome (disease) variable.

The chromium level of 1.4 mcg/L was set as the standpoint between normal and abnormal chromium levels. The albumin creatinine ratio of 300 mg/g was set as the standpoint between normal and albuminuria (micro and macro). Both micro-albuminuria and macro-albuminuria were categorized in the single ‘albuminuria’ category. Glycohemoglobin level was used as a continuous variable without conversion to a categorical variable. Because of possible confounding due to diabetes treatment (glucose-lowering medications), all respondents with the ‘yes’ answer to the question ‘take diabetic pills to lower blood sugar’ were excluded from the analysis.

### The aggravating (positive) interaction effect between blood cobalt level and old age on albuminuria (micro and macro)

The laboratory data and demographics data of NHANES 2015–2016 and NHANES 2017–2018 datasets were used. The blood cobalt level (mcg/L) in laboratory data was used as the environmental exposure variable, and age in years in demographics data was used as the susceptibility factor variable. Albumin creatinine ratio (mg/g) in laboratory data was used as the outcome variable.

The cobalt level of 1.8 mcg/L was set as the standpoint between normal and abnormal cobalt levels. The albumin creatinine ratio of 300 mg/g was set as the standpoint between normal and albuminuria. Both micro-albuminuria and macro-albuminuria were categorized as a single ‘albuminuria’ category. Age in years was applied as a continuous variable without conversion to a categorical variable.

### Calculation of estimates

All abbreviations used in this article are provided in Table 1. First, the estimate with an appropriate confidence interval for the fold-difference in the odds of albuminuria associated with a unit difference in the blood chromium level was calculated in the first example. In the second example, the estimate with an appropriate confidence interval for the fold-difference in the odds of albuminuria associated with a unit difference in the blood cobalt level was calculated. Second, the estimate with an appropriate confidence interval for the fold-difference in the odds of albuminuria associated with a unit difference in the blood glycohemoglobin level was calculated in the first example. In the second example, the estimate with an appropriate confidence interval for the fold-difference in the odds of albuminuria associated with a unit difference in the age in years was calculated. Third, the estimate with an appropriate confidence interval for the multiplicative ICR associated with the difference of one unit in both the blood chromium level and the blood glycohemoglobin level was calculated in the first example. In the second example, the estimate with an appropriate confidence interval for the multiplicative ICR associated with the difference of one unit in both the blood cobalt level and age in years was calculated. Fourth, the independence between the blood chromium level and blood glycohemoglobin level was assessed in the whole sample, including cases and non-cases in the first example. In the second example, the independence between the blood cobalt level and age in years was assessed in the whole sample, including cases and non-cases. Fifth, only if the independence mentioned in the fourth item was plausible the multiplicative ICR using only cases were calculated. If the independence mentioned in the fourth item was not plausible, the multiplicative ICR calculated based on only cases was adjusted based on theoretical equations (multiplied by the S-E OR_c/nc_). After these steps, the authors concluded whether the estimate derived from only cases is more precise than the estimate obtained from both cases and non-cases.

**Table 1:** Abbreviations

Abbreviations	Definition	Equation
RR	Relative Risk	
OR	Odds Ratio	
S	Susceptibility factor	
E	Environmental exposure	
ICR_c/nc_	The interaction contrast ratio (ICR) in a study with cases and non-cases	${\mathrm{ICR}}_{\mathrm{c}/\mathrm{nc}}=\frac{{\mathrm{RR}}_{\mathrm{s}\mathrm{e}}}{{\mathrm{RR}}_{\mathrm{s}}{\mathrm{RR}}_{\mathrm{e}}}=\left(\frac{\mathrm{ag}}{\mathrm{c}\mathrm{e}}\right)\left(\frac{\left(\mathrm{c}+\mathrm{D}\right)\left(\mathrm{e}+\mathrm{F}\right)}{\left(\mathrm{a}+\mathrm{B}\right)\left(\mathrm{g}+\mathrm{H}\right)}\right)$
ICR_cc_	The ICR in a case-control study	${\mathrm{ICR}}_{\mathrm{cc}}=\frac{{\mathrm{OR}}_{\mathrm{s}\mathrm{e}}}{{\mathrm{OR}}_{\mathrm{s}}{\mathrm{OR}}_{\mathrm{e}}}=\left(\frac{\mathrm{ag}}{\mathrm{ce}}\right)\left(\frac{\mathrm{DF}}{\mathrm{BH}}\right)$
ICR_co_	The ICR in a case-only study	${\mathrm{ICR}}_{\mathrm{co}}=\left(\frac{\mathrm{ag}}{\mathrm{ce}}\right)$
S-E OR_c/nc_	Susceptibility factor-Environmental exposure odds ratio in a study with cases and non-cases	S-E OR_c/nc_ = $\left(\frac{\left(\mathrm{c}+\mathrm{D}\right)\left(\mathrm{e}+\mathrm{F}\right)}{\left(\mathrm{a}+\mathrm{B}\right)\left(\mathrm{g}+\mathrm{H}\right)}\right)$
S-E OR_control_	Susceptibility factor-Environmental exposure odds ratio in the control population	S-E OR_control_$=\left(\frac{\mathrm{DF}}{\mathrm{BH}}\right)=\frac{\mathrm{df}}{\mathrm{bh}}$

### Statistical method and software

A logistic regression model was applied for the calculation of odds ratios. The R software version 4.0.3 was used. Package ‘dplyr’ and ‘data.table’ were used for the pre-processing of the datasets. The used R codes are provided in Supplementary material A.

## Methods: theoretical backgrounds

### Basic assumption: the joint and ICR on the multiplicative scale

Statistical interactions between the effects of susceptibility factors and those of environmental factors can be assessed as departures from multiplicativity of effects or as departures from additivity of effects. Table 2 indicates an example of a study with cases and non-cases. With the unexposed and no susceptibility (E-G-) group set as the reference group, we can calculate relative risk (RR) and odds ratio (OR) for all other 3 groups.

**Table 2:** An example of a study with cases and non-cases

Environment (E)	Susceptibility (S)	Disease	No disease	Total	Relative Risk (RR)	Odds Ratio (OR)
–	–	a	B	a + B	1.0 (ref)	1.0 (reference)
–	+	c	D	c + D	RR_s_=$\frac{\mathrm{c}\left(\mathrm{a}+\mathrm{B}\right)}{\mathrm{a}\left(\mathrm{c}+\mathrm{D}\right)}$	OR_s_=$\frac{\mathrm{cB}}{\mathrm{aD}}$
+	–	e	F	e + F	RR_e_=$\frac{\mathrm{e}\left(\mathrm{a}+\mathrm{B}\right)}{\mathrm{a}\left(\mathrm{e}+\mathrm{F}\right)}$	OR_e_=$\frac{\mathrm{eB}}{\mathrm{aF}}$
+	+	g	H	g + H	RR_se=_$\frac{\mathrm{g}\left(\mathrm{a}+\mathrm{B}\right)}{\mathrm{a}\left(\mathrm{g}+\mathrm{H}\right)}$	OR_se=_$\frac{\mathrm{gB}}{\mathrm{aH}}$

Under additive scale: ICR_c/nc_ = RR_se_-(RR_s_ + RR_e_-1), ICR_c/nc_ = OR_se_-(OR_s_ + OR_e_-1)

Under multiplicative scale: ICR_c/nc_ = RR_se_/(RR_s_ × RR_e_), ICR_c/nc_ = OR_se_/(OR_s_ × OR_e_)

The joint RR for the susceptibility factor and environmental exposure (RR_se_) can be compared with the RR for environmental exposure alone (RR_e_) or with the RR for susceptibility factor alone (RR_s_). The joint OR for the susceptibility factor and environmental exposure (OR_se_) can be compared with the OR for environmental exposure alone (OR_e_) or with the OR for susceptibility factor alone (OR_s_). In the joint RR model with the additive scale, the ICR (ICR_c/nc_) indicates the departures from the sum of individual RRs minus one (ICR_c/nc_ = RR_se_-(RR_s_ + RR_e_-1)). This equation is called ‘relative excess risk due to interaction (RERI)’ in epidemiologic literature [15]. In the joint OR model with the additive scale, the ICR (ICR_c/nc_) indicates the departures from the sum of individual ORs minus one (ICR_c/nc_ = OR_se_-(OR_s_ + OR_e_-1)). In the joint RR model with the multiplicative scale, the ICR (ICR_c/nc_) indicates the departures from the product of individual RRs (ICR_c/nc_ = RR_se_/(RR_s_ × RR_e_)). In the joint OR model with the multiplicative scale, the ICR (ICR_c/nc_) indicates the departures from the product of individual ORs (ICR_c/nc_ = OR_se_/(OR_s_ × OR_e_)). In this article, we used only the joint RR or the joint OR model with the multiplicative scale to estimate the ICR_c/nc_.

### The ICR in a case-only study and the ICR in a study with cases and non-cases

Table 2 illustrates the composition of a study with cases and non-cases. To generate case-only data from the above source population, we extracted only the ‘case’ column in Table 3.

**Table 3:** An example of a case-only study

		Susceptibility (S)	
		–	+
Environment (E)	**–**	a	c
	**+**	e	g

The ICR in a case-only study will be as follows:

1 $${\mathrm{ICR}}_{\mathrm{c}\mathrm{o}}=\frac{\left[\frac{\left\{\frac{\mathrm{a}}{\mathrm{a}+\mathrm{e}}\right\}}{\left\{\frac{\mathrm{e}}{\mathrm{a}+\mathrm{e}}\right\}}\right]}{\left[\frac{\left\{\frac{\mathrm{c}}{\mathrm{c}+\mathrm{g}}\right\}}{\left\{\frac{\mathrm{g}}{\mathrm{c}+\mathrm{g}}\right\}}\right]}=\left(\frac{\mathrm{a}\mathrm{g}}{\mathrm{c}\mathrm{e}}\right)$$

The ICR in a study with cases and non-cases will be as follows:

2 $$\kern1em {\mathrm{ICR}}_{\mathrm{c}/\mathrm{nc}}=\frac{{\mathrm{RR}}_{\mathrm{s}\mathrm{e}}}{{\mathrm{RR}}_{\mathrm{s}}{\mathrm{RR}}_{\mathrm{e}}}=\left(\frac{\mathrm{ag}}{\mathrm{c}\mathrm{e}}\right)\left(\frac{\left(\mathrm{c}+\mathrm{D}\right)\left(\mathrm{e}+\mathrm{F}\right)}{\left(\mathrm{a}+\mathrm{B}\right)\left(\mathrm{g}+\mathrm{H}\right)}\right)=\left({\mathrm{ICR}}_{\mathrm{c}\mathrm{o}}\right)\left(\frac{\left(\mathrm{c}+\mathrm{D}\right)\left(\mathrm{e}+\mathrm{F}\right)}{\left(\mathrm{a}+\mathrm{B}\right)\left(\mathrm{g}+\mathrm{H}\right)}\right)$$

In Eq. (2), (ag/ce) is converted into ICR_co_ obtained in the case-only study. ICR_c/nc_ is the ICR calculated in a study with cases and non-cases. From Eq. (2), the requirement for the equality between the ICR acquired from a study with cases and non-cases and the ICR acquired from the case-only study is as follows:

3 $$\left(\frac{\left(\mathrm{c}+\mathrm{D}\right)\left(\mathrm{e}+\mathrm{F}\right)}{\left(\mathrm{a}+\mathrm{B}\right)\left(\mathrm{g}+\mathrm{H}\right)}\right)=\mathrm{S}-\mathrm{E}\ {\mathrm{OR}}_{\mathrm{c}/\mathrm{nc}}=1$$

Equation (3) means that the environmental exposure and the susceptibility factor must be independent in a study with cases and non-cases for the equality between the ICR acquired from a study with cases and non-cases and the ICR acquired from the case-only study. In Eqs. (2) and (3), we should note that the equality between the ICR from a study with case and non-cases and the ICR from the case-only study does not necessarily require a rare disease assumption (a low prevalence of the disease).

The above equations in this subsection can be understood from the context of a logistic model, with other covariates adjusted. The following equations indicate a conventional logistic regression model for a case-only study:

4 $$\mathrm{logit}\ \mathrm{P}\left(\mathrm{S}=1\right)={\upgamma}_0+{\upgamma}_1\ \mathrm{E}$$

5 $${\mathrm{ICR}}_{\mathrm{co}}=\exp \left({\upgamma}_1\right)$$

When E is a categorical or continuous variable for environmental exposure status, a case-only estimate for the interaction effect can be obtained using Eq. (5).

We can also assess the independence between an environmental factor and a susceptibility factor in a study with cases and non-cases from the context of a logistic model using the following equations:

6 $$\mathrm{logit}\ \mathrm{P}\left(\mathrm{S}=1\right)={\upeta}_0+{\upeta}_1\mathrm{E}$$

7 $$\mathrm{S}-\mathrm{E}\ {\mathrm{OR}}_{\mathrm{c}/\mathrm{nc}}=\exp \left({\upeta}_1\right)$$

According to the independence assumption provided in Eq. (3), the environmental exposure and the susceptibility factor must be independent in the population with cases and non-cases for the equality between the ICR obtained in the population with cases, and non-cases and the ICR obtained in the case-only study. From the context of a logistic model, this means that the confidence interval for Eq. (7) must include 1 and that the point estimate for Eq. (7) must be close to 1.

We can also calculate the ICR obtained in the population with cases and non-cases from the context of a logistic model, using the following equation:

8 $$\mathrm{logit}\ \mathrm{P}\left(\mathrm{D}=1\right)={\upbeta}_0+{\upbeta}_1\mathrm{S}+{\upbeta}_2\mathrm{E}+{\upbeta}_3\mathrm{SE}$$

9 $${\mathrm{ICR}}_{\mathrm{c}/\mathrm{nc}}=\exp \left({\upbeta}_3\right)$$

### The ICR in a case-control study

We can define the susceptibility-environment ICR acquired from a case-control study in the model with the multiplicative scale as follows:

10 $${\mathrm{ICR}}_{\mathrm{cc}}={\mathrm{OR}}_{\mathrm{s}\mathrm{e}}/\left({\mathrm{OR}}_{\mathrm{s}}\times {\mathrm{OR}}_{\mathrm{e}}\right)$$

ICR_cc_: the ICR calculated in a case-control study.

ICR_cc_ > 1: The joint OR is larger than the product of each individual OR.

ICR_cc_ < 1: The joint OR is smaller than the product of each individual OR.

ICR_cc_ = 1: The joint OR is the same as the product of each individual OR.

If the joint OR is larger than the product of each individual OR, the ICR_cc_ will be larger than 1. If the joint OR is smaller than the product of each individual OR, the ICR_cc_ will be smaller than 1. If the joint OR is the same as the product of each individual OR, the ICR_cc_ will be 1.

### The ICR in a case-only study and the ICR in a case-control study

For the generation of the case-control study data, a fraction (p) of controls in each group was selected from the population with cases and non-cases in Table 4.

**Table 4:** A case-control study data generated from a population with cases and non-cases

Environment (E)	Susceptibility (S)	Case	Control	Odds Ratio (OR)
–	–	a	b = pB	1.0 (ref)
–	+	c	d = pD	OR_s_=$\frac{\mathrm{cb}}{\mathrm{ad}}$=$\frac{\mathrm{cB}}{\mathrm{aD}}$
+	–	e	f = pF	OR_e_=$\frac{\mathrm{eb}}{\mathrm{af}}$=$\frac{\mathrm{eB}}{\mathrm{aF}}$
+	+	g	h = pH	OR_se_=$\frac{\mathrm{gb}}{\mathrm{ah}}$=$\frac{\mathrm{gB}}{\mathrm{aH}}$

The ICR in a case-control study can be calculated as follows:

11 $${\mathrm{ICR}}_{\mathrm{cc}}=\frac{{\mathrm{OR}}_{\mathrm{s}\mathrm{e}}}{{\mathrm{OR}}_{\mathrm{s}}{\mathrm{OR}}_{\mathrm{e}}}=\left(\frac{\mathrm{ag}}{\mathrm{ce}}\right)\left(\frac{\mathrm{DF}}{\mathrm{BH}}\right)=\left({\mathrm{ICR}}_{\mathrm{co}}\right)\left(\frac{\mathrm{DF}}{\mathrm{BH}}\right)$$

In Eq. (11), the requirement for equality between ICR_cc_ and ICR_co_ is as follows:

12 $$\left(\frac{\mathrm{DF}}{\mathrm{BH}}\right)=\frac{\mathrm{df}}{\mathrm{bh}}=\mathrm{S}-\mathrm{E}\ {\mathrm{OR}}_{\mathrm{control}}=1$$

Equation (12) means that for the equality between ICR_cc_ and ICR_co_, the susceptibility factor and environmental exposure must be independent in the control population. A rare disease assumption is also not required for this equality.

We can also calculate the ICR in a case-control study from the context of a logistic model, using the following equation:

13 $$\mathrm{logit}\ \mathrm{P}\left(\mathrm{D}=1\right)={\upbeta}_0+{\upbeta}_1\mathrm{S}+{\upbeta}_2\mathrm{E}+{\upbeta}_3\mathrm{SE}$$

14 $${\mathrm{ICR}}_{\mathrm{cc}}=\exp \left({\upbeta}_3\right)$$

### The ICR in a study with cases and non-cases and the ICR in a case-control study

The equality between ICR_cc_ and ICR_co_ does not mean that these 2 estimates are not biased away from the ICR acquired from the population with cases and non-cases (ICR_c/nc_). Based on Eqs. (2) and (11), we can get the following equation:

15 $${\mathrm{ICR}}_{\mathrm{c}\mathrm{c}}={\mathrm{ICR}}_{\mathrm{c}/\mathrm{nc}}\ \frac{\left(\mathrm{DF}\right)}{\left(\mathrm{BH}\right)}\ \left(\frac{\left(\mathrm{a}+\mathrm{B}\right)\left(\mathrm{g}+\mathrm{H}\right)}{\left(\mathrm{c}+\mathrm{D}\right)\left(\mathrm{e}+\mathrm{F}\right)}\right)$$

In Eq. (15), for the equality between ICR_cc_ and ICR_c/nc_, the following equation or at least 1 of 2 conditions suggested below should be met:

16 $${\displaystyle \begin{array}{c}\frac{\left(\mathrm{DF}\right)}{\left(\mathrm{BH}\right)}\ \left(\frac{\left(\mathrm{a}+\mathrm{B}\right)\left(\mathrm{g}+\mathrm{H}\right)}{\left(\mathrm{c}+\mathrm{D}\right)\left(\mathrm{e}+\mathrm{F}\right)}\right)=1\\ {}\left[\mathrm{S}-\mathrm{E}\ {\mathrm{OR}}_{\mathrm{c}\mathrm{ontrol}}=\mathrm{S}-\mathrm{E}\ {\mathrm{OR}}_{\mathrm{c}/\mathrm{nc}}=1\right]\ \mathrm{or}\ \left[\mathrm{the}\ \mathrm{disease}\ \mathrm{is}\ \mathrm{rare}\right]\end{array}}$$

Equation (16) means that for the equality between ICR_cc_ and ICR_c/nc_, the susceptibility factor and the environmental exposure must be independent both in the population with cases and non-cases and in the controls. Alternatively, if the disease is rare, Eq. (16) will be satisfied. In this case, the rare disease assumption must be examined in the population with cases and non-cases.

### S-E independence in the population with cases and non-cases and S-E independence in the controls: one cannot replace the other

If we evaluate Eq. (16) in detail, we can find an important relationship. The S-E independence in the controls is a totally different concept from the S-E independence in the population with cases and non-cases: one cannot replace the other.

17 $${\mathrm{ICR}}_{\mathrm{c}\mathrm{c}}={\mathrm{ICR}}_{\mathrm{c}\mathrm{o}}={\mathrm{ICR}}_{\mathrm{c}/\mathrm{nc}}$$

For the first equal sign, S-E OR_control_ = 1 is required according to Eq. (11).

For the second equal sign, S-E OR_c/nc_ = 1 is required according to Eq. (2).

If the disease is rare, ${\mathrm{ICR}}_{\mathrm{cc}}=\left({\mathrm{ICR}}_{\mathrm{co}}\right)\left(\frac{\mathrm{DF}}{\mathrm{BH}}\right)$ according to Eq. (11), and ${\mathrm{ICR}}_{\mathrm{c}/\mathrm{nc}}=\left({\mathrm{ICR}}_{\mathrm{c}\mathrm{o}}\right)\left(\frac{\mathrm{DF}}{\mathrm{BH}}\right)$ according to Eq. (2).

18 $$\mathrm{Therefore},\kern0.5em {\mathrm{ICR}}_{\mathrm{c}\mathrm{c}}={\mathrm{ICR}}_{\mathrm{c}/\mathrm{nc}}\ne {\mathrm{ICR}}_{\mathrm{c}\mathrm{o}}$$

If a researcher uses whether or not S-E OR_controls_ equals 1, instead of whether or not S-E OR_c/nc_ equals 1, for the assessment of the validity of using ICR_co_ instead of using ICR_c/nc_, this misuse can lead to either the rejection of the valid ICR_co_ or the acceptance of the invalid ICR_co_ mistakenly.

In Supplementary material B, an example from Gatto et al. [8] is provided for this problem. In the first example, S and E are independent in the population, including cases and non-cases (S-E OR_c/nc_ = 1). The interaction estimate in the population, including cases and non-cases (i.e., ICR_c/nc_) is 2.5. The ICR_co_ is also 2.5. In this situation, the S-E OR_control_ of 0.7 does not provide a reliable estimation for S-E OR_c/nc_ of 1.0. In the second example, the S-E OR_c/nc_ is 2.0, showing a non-independent relationship. The ICR_c/nc_ is 1.0, but ICR_co_ is 2.0. In this situation, the S-E OR_control_ of 1.0 does not provide a reliable estimation for S-E OR_c/nc_ of 2.0.

### The rare disease assumption: for ICR_cc_ = ICR_c/nc_ and S-E OR_control_ = S-E OR_c/nc_

The rare disease assumption provides 2 implications in this discussion of the case-only approach. The first implication is provided in Eq. (18). The second implication is the following:

$\left(\frac{\left(\mathrm{c}+\mathrm{D}\right)\left(\mathrm{e}+\mathrm{F}\right)}{\left(\mathrm{a}+\mathrm{B}\right)\left(\mathrm{g}+\mathrm{H}\right)}\right)=$S-E OR_c/nc_ from Eq. (3) $\mathrm{and}\ \left(\frac{\mathrm{DF}}{\mathrm{BH}}\right)=\frac{\mathrm{df}}{\mathrm{bh}}=$ S-E OR_control_ from Eq. (12)

19 $$\mathrm{If}\ \mathrm{the}\ \mathrm{disease}\ \mathrm{is}\ \mathrm{rare},\mathrm{S}-\mathrm{E}\ {\mathrm{OR}}_{\mathrm{c}/\mathrm{nc}}=\mathrm{S}-\mathrm{E}\ {\mathrm{OR}}_{\mathrm{c}\mathrm{ontrol}}=\left(\frac{\mathrm{DF}}{\mathrm{BH}}\right)$$

In this subsection, we will deal with the second implication. Equation (20) indicates the relationship between S-E OR_control_ and S-E OR_c/nc_ [8].

20 $$\mathrm{S}-\mathrm{E}\ {\mathrm{OR}}_{\mathrm{c}\mathrm{ontrol}}=\mathrm{S}-\mathrm{E}\ {\mathrm{OR}}_{\mathrm{c}/\mathrm{nc}}\times \left(\frac{\left(\frac{1}{\mathrm{p}\left(\mathrm{D}|\mathrm{S}-\mathrm{E}-\right)}-1\right)\times \left(\frac{1}{\mathrm{p}\left(\mathrm{D}|\mathrm{S}-\mathrm{E}-\right)}-{\mathrm{RR}}_{\mathrm{SE}}\right)}{\left(\frac{1}{\mathrm{p}\left(\mathrm{D}|\mathrm{S}-\mathrm{E}-\right)}-{\mathrm{RR}}_{\mathrm{G}}\right)\times \left(\frac{1}{\mathrm{p}\left(\mathrm{D}|\mathrm{S}-\mathrm{E}-\right)}-{\mathrm{RR}}_{\mathrm{E}}\right)}\right)$$

In Gatto et al. [8], the authors used Eq. (20) to conduct a sensitivity analysis (Supplementary material C). The article assessed the impact of the baseline risk of disease in the population (p(D|S-E-)) and the independent effect of S (RR_S_) on the S-E OR_control_ when the S-E OR_c/nc_ is 1.0. In Supplementary material C, the baseline risk of disease ranges from 0.1 to 6%. As illustrated in Supplementary material C, the S-E OR_control_ is similar to the S-E OR_c/nc_ of 1.0 when either the baseline risk of disease (p(D|S-E-)) is under 1%, and the independent effect of S is relatively low (RR_S_ < 2.5). However, as the baseline risk of disease approaches 3%, the S-E OR_control_ begins to diverge from the S-E OR_c/nc_ of 1.0. This worsens when the independent effect of the susceptibility factor increases.

### Violation of independence: confounder and subpopulation dependence

The violation of independence between S and E occurs when an individual alters his or her environmental exposure according to his or her susceptibility factor. This violation is due to 2 factors mainly: (i) a confounder and (ii) subpopulation dependence.

Gatto et al. [8] provide 2 examples of confounders. In the first example of Supplementary material D, the family history functions as a confounder, and in the second example of Supplementary material D, the adverse reaction to alcohol functions as a mediator between the susceptibility factor and the environmental exposure. For these 2 examples, the positive multiplicative interaction (ICR_CO_ of > 1) will be biased towards the null (ICR_CO_ ≈ 1) because of the overall negative association between S and E due to C.

If these covariates can be adjusted, the independence between S and E can be restored.

21 $$\mathrm{logit}\ \mathrm{P}\left(\mathrm{S}=1\right)={\upgamma_0}^{'}+{\upgamma_1}^{'}\mathrm{E}+{\upgamma_2}^{'}\mathrm{C}$$

22 $$\mathrm{adjusted}\ {\mathrm{ICR}}_{\mathrm{CO}}\left(\mathrm{adjusted}\ \mathrm{for}\ \mathrm{covariate}\ \mathrm{C}\right)=\exp \left({\upgamma_1}^{'}\right)$$

However, a cautious approach is required because the adjustment of unrelated covariates with S-E dependence would cost some degrees of freedom and would reduce the precision of ICR_CO_ [8].

Another source of the violation of independence is a hidden dependence on a subpopulation. Wang et al. [9] provide a unique solution for this problem, providing the following Eq. (9):

23 $$\mathrm{CIR}={\mathrm{r}}_{\mathrm{S}\mathrm{E}}\ \mathrm{\times}\ {\mathrm{CV}}_{\mathrm{S}}\ \mathrm{\times}\ {\mathrm{CV}}_{\mathrm{E}}+1$$

CIR: Confounding Interaction Ratio. r_SE_: the correlation coefficient between S and E. CV_S_: variation in susceptibility factor prevalence odds. CV_E_: variation in environmental exposure prevalence odds.

24 $${\mathrm{CIR}}_{\mathrm{U}}=\frac{\sqrt{\upupsilon_{\mathrm{S}}{\upupsilon}_{\mathrm{E}}}\times {\left(\sqrt{\upupsilon_{\mathrm{S}}{\upupsilon}_{\mathrm{E}}}+1\right)}^2}{\left(\sqrt{\upupsilon_{\mathrm{S}}{\upupsilon}_{\mathrm{E}}}+{\upupsilon}_{\mathrm{S}}\right)\left(\sqrt{\upupsilon_{\mathrm{S}}{\upupsilon}_{\mathrm{E}}}+{\upupsilon}_{\mathrm{E}}\right)}\ge 1,{\mathrm{CIR}}_{\mathrm{L}}=\frac{1}{\mathrm{U}}\le 1$$

CIR_U_: the upper bound of CIR, CIR_L_: the lower bound of CIR, *υ*_*S*_(*υ*_*S*_ ≥ 1): the ratio of the largest and the smallest susceptibility frequency odds across all strata. *υ*_*E*_(*υ*_*E*_ ≥ 1): the ratio of the largest and the smallest exposure frequency odds across all strata.

In Eq. (23), CIR is the ratio of the crude ICR_c/nc_ without stratification over ICR_c/nc_ with stratification. According to the above equation, there would be no population stratification bias (CIR =1), (i) if the exposure prevalence odds and the susceptibility frequency odds are uncorrelated across all strata (r_ES_ = 0), (ii) no variation exists in the exposure prevalence odds (CV_E_ = 0), or (iii) no variation exists in the susceptibility frequency odds (CV_S_ = 0).

In Eq. (24), *υ*_*S*_(*υ*_*S*_ ≥ 1) denotes the ratio of the largest over the smallest susceptibility frequency odds, and *υ*_*E*_(*υ*_*E*_ ≥ 1) denotes the ratio of the largest over the smallest exposure prevalence odds across all the strata in the population. If there is either no variation in the susceptibility frequency odds (*υ*_*S*_ = 1) or in the exposure prevalence odds (*υ*_*E*_ = 1), there would be no bias (U = L = 1) according to Eq. (24). If we can calculate CIR for a population, we can calculate ICR_c/nc_ with stratification.

For the violation of S-E independence, researchers usually would try to evaluate a potential confounder based on their subject-matter knowledge. However, for subpopulation dependence, attention should be paid to the whole study population and the strata rather than finding a confounder. This important difference should be in the mind of researchers using a case-only approach.

### The efficiency gained from the case-only approach

Case-only approach can calculate a more precise interaction effect estimate (i.e., that with a narrower confidence interval) than a study design with case and non-cases, such as a cohort/case-control study approach can do [16].

In Eqs. (8) and (9), and Table 2, the asymptotic variance of $\hat{\upbeta}$_3_ in a population with cases and non-cases is as follows:

25 $$\mathrm{Var}\left({\hat{\upbeta}}_3\right)=\frac{1}{a}+\frac{1}{B}+\frac{1}{c}+\frac{1}{D}+\frac{1}{e}+\frac{1}{F}+\frac{1}{g}+\frac{1}{H}$$

In Eqs. (13) and (14), and Table 4, the asymptotic variance of ${\overline{\overline{\upbeta}}}_3$ in a case-control study is as follows:

26 $$\mathrm{Var}\left(\overline{\overline{\upbeta_3}}\right)=\frac{1}{a}+\frac{1}{b}+\frac{1}{c}+\frac{1}{d}+\frac{1}{e}+\frac{1}{f}+\frac{1}{g}+\frac{1}{h}$$

In Eqs. (4), Eq. (5), and Table 3, the asymptotic variance of $\hat{\gamma}$_1_ in a case-only study is as follows:

27 $$\mathrm{Var}\left({\hat{y}}_1\right)=\frac{1}{a}+\frac{1}{c}+\frac{1}{e}+\frac{1}{g}$$

Comparing Eq. (27) with Eqs. (25) and (26), the case-only design can provide an estimate with a narrower confidence interval than either the case-control or the cohort design (study designs with cases and non-cases) can do. This efficiency gain comes from the independence assumption between susceptibility factor and environmental exposure (S-E OR_c/nc_ = 1).

### Methodological issues to be considered

Several issues must be considered when applying the case-only approach to estimating the interaction effect between a susceptibility factor and an environmental exposure. Firstly, the case selection process must follow a typical rule of case selection as in a case-control study. Secondly, researchers must verify independence between the susceptibility trait and the environmental exposure in the population with cases and non-cases to substitute the ICR_CO_ calculated in a case-only design for the ICR_c/nc_ calculated in a population with cases and non-cases (according to Eqs. (2) and (3)). If evidence of an association between susceptibility factor and environmental exposure exists, the calculated S-E OR_c/nc_ must be used to correct the ICR_CO_ by multiplying it as provided in Eq. (2). Thirdly, the independence assumption might seem reasonable for various susceptibility factors and environmental exposures. However, some susceptibility factors can modify the likelihood of environmental exposure. This hidden association must be discovered before a case-only approach is applied. Finally, the interaction effect estimate (ICR_CO_) obtained from the case-only approach can only be interpreted as a departure from the multiplicative effect and not from the additive effect. However, according to previous epidemiologic literature, additive interaction more closely corresponds to mechanistic biologic interaction effects rather than merely statistical interaction effects [17, 18]. Even though this is true, researchers in the current academic societies often use the multiplicative scale to estimate interaction effects because of several practical reasons [18]. This limitation should be considered when the results of this study are applied.

### Summary

In summary, the case-only approach can be applied to environmental epidemiology successfully when a susceptibility factor and an environmental exposure are independent in a population with cases and non-cases. Through this approach, a more precise interaction effect estimate can be calculated.

## Results

### Basic information of datasets and descriptive analysis for each variable

By combining ‘Albumin & Creatinine – Urine,’ ‘Chromium & Cobalt,’ ‘Glycohemoglobin,’ and ‘Demographic Variables and Sample Weights’ data files, a dataset with 7286 subjects was created. For the first analysis example, the respondents with the ‘yes’ answer to the question ‘take diabetic pills to lower blood sugar’ were excluded (5890 subjects). After that, only 1396 subjects were included. For the second analysis example, all subjects (7286 subjects created) were included. The descriptive analysis results for the main variables are provided in Table 5.

**Table 5:** Descriptive analysis for each variable used

The preventive (negative) interaction effect between blood chromium levels and glycohemoglobin levels on albuminuria (micro and macro)				
Environmental exposure	Normal chromium	Abnormal chromium		NA
Number of subjects	1312	29		55
Mean	0.35 μg/L	2.02 μg/L		NA
Outcome (disease)	No albuminuria	Albuminuria		NA
Number of subjects	1089	270		37
Mean	9.49 mg/g	504.0 mg/g		NA
Susceptibility factor	Blood glycohemoglobin level (with 48 NA values)			
Statistics	Min	Median	Mean	Max
Value	4.1%	6.0%	6.39%	16.5%
The aggravating (positive) interaction effect between blood cobalt levels and old ages on albuminuria (micro and macro)				
Environmental exposure	Normal cobalt	Abnormal cobalt		NA
Number of subjects	6942	32		312
Mean	0.18 μg/L	6.04 μg/L		NA
Outcome (disease)	No albuminuria	Albuminuria		NA
Number of subjects	5919	1179		188
Mean	9.28 mg/g	335.0 mg/g		NA
Susceptibility factor	Age in years (with no NA value)			
Statistics	Min	Median	Mean	Max
Value	40.0 yrs	60.0 yrs	60.28 yrs	80.0 yrs

*NA* not available (missing value), *yrs* years of age

### The negative interaction effect between blood chromium level and glycohemoglobin level on albuminuria (micro and macro)

As the first example, Table 6 provides the sequential processes of applying the case-only approach (which will be explained in the first discussion section) in estimating the interaction effect between blood chromium level and glycohemoglobin level on albuminuria. All these sequential processes follow the sequential processes provided in subsection 2.3: (i) Firstly, a 1 μg/L difference of blood chromium level resulted in the fold-difference in the odds of albuminuria 2.20 (95% CI 1.48–3.32) times. (ii) Secondly, a 1% difference in blood glycohemoglobin level resulted in the fold-difference in the odds of albuminuria 1.57 (95% CI 1.44–1.73) times. (iii) Thirdly, when a 1 μg/L difference in blood chromium level and a 1% difference in blood glycohemoglobin level coincide, the multiplicative interaction contrast ratio (ICR) is 0.72 (95% CI 0.35–1.60), with statistical insignificance. (iv) Fourthly, in the population with cases and non-cases, blood chromium levels and blood glycohemoglobin levels are independent of each other (S-E OR_c/nc_: 0.76 (95% CI 0.47–1.06)). Therefore, the case-only ICR can be a good substitute for the ICR acquired from the population with cases and non-cases. (v) Finally, when only the cases are analyzed (case-only approach), the case-only ICR is 0.59 (95% CI 0.28–0.95), with a statistical significance (a negative interaction effect).

**Table 6:** The application of the case-only approach for the first and second example

Table 6-1. The application of the case-only approach for the preventive (negative) interaction effect between blood chromium levels and glycohemoglobin levels on albuminuria (micro and macro)	
logit P(D = 1) = β_0_ + β_2_’E OR for 1 unit difference of environmental exposure = exp.(β_2_’)	
OR for a 1 μg/L difference of blood chromium level: 2.20 (95% CI 1.48–3.32)	Effect estimate
When a 1 μg/L of blood chromium level (μg/L) differs, the fold-difference in the odds of albuminuria is 2.20 (95% CI 1.48–3.32) times.	Explanation
logit P(D = 1) = β_0_ + β_1_’S OR for 1 unit difference of susceptibility factor = exp.(β_1_’)	
OR for 1% difference of glycohemoglobin level: 1.57 (95% CI 1.44–1.73)	Effect estimate
When a 1% of blood glycohemoglobin level differs, the fold-difference in the odds of albuminuria is 1.57 (95% CI 1.44–1.73) times.	Explanation
logit P(D = 1) = β_0_ + β_1_S + β_2_E + β_3_SE ICR_c/nc_ = exp.(β_3_)	Eq. (8) Eq. (9)
ICR_c/nc_: 0.72 (95% CI 0.35–1.60)	Effect estimate
When a 1 μg/L of both blood chromium level and 1% of blood glycohemoglobin level coincide, the multiplicative ICR is 0.72 (95% CI 0.35–1.60), with statistical insignificance.	Explanation
logit P(S = 1) = η_0_ + η_1_E S-E OR_c/nc_ = exp.(η_1_)	Eq. (6) Eq. (7)
S-E OR_c/nc_: 0.76 (95% CI 0.47–1.06)	Effect estimate
In the the population with cases and non-cases, blood chromium levels and blood glycohemoglobin levels are independent. Therefore, the case-only ICR can be a good substitute for the ICR acquired from the population with cases and non-cases.	Explanation
logit P(S = 1) = γ_0_ + γ_1_E ICR_CO_ = exp.(γ_1_)	Eq. (4) Eq. (5)
ICR_CO_: 0.59 (95% CI 0.28–0.95)	Effect estimate
When only the cases are analyzed (case-only approach), the case-only ICR is 0.59 (95% CI 0.28–0.95), with a statistical significance (a negative interaction effect).	Explanation
Table 6-2. The application of the case-only approach for the aggravating (positive) interaction effect between blood cobalt levels and old ages on albuminuria (micro and macro)	
logit P(D = 1) = β_0_ + β_2_’E OR for 1 unit difference of environmental exposure = exp.(β_2_’)	
OR for 1 μg/L difference of blood cobalt level: 1.09 (95% CI 0.98–1.20)	Effect estimate
When a 1 μg/L of blood cobalt level (μg/L) differs, the fold-difference in the odds of albuminuria is 1.09 (95% CI 1.31–1.57) times.	Explanation
logit P(D = 1) = β_0_ + β_1_’S OR for 1 unit difference of susceptibility factor = exp.(β_1_’)	
OR for a 1-year difference of age: 1.05 (95% CI 1.04–1.05)	Effect estimate
When 1-year in age differs, the fold-difference in the odds of albuminuria is 1.05 (95% CI 1.04–1.05) times.	Explanation
logit P(D = 1) = β_0_ + β_1_S + β_2_E + β_3_SE ICR_c/nc_ = exp.(β_3_)	Eq. (8) Eq. (9)
ICR_c/nc_: 1.13 (95% CI 0.99–1.37)	Effect estimate
When a 1 μg/L difference of both blood cobalt level and 1-year difference of age coincide, the multiplicative ICR is 1.13 (95% CI 0.99–1.37), with statistical insignificance.	Explanation
logit P(S = 1) = η_0_ + η_1_E S-E OR_c/nc_ = exp.(η_1_)	Eq. (6) Eq. (7)
S-E OR_c/nc_: 1.06 (95% CI 1.03–1.10)	Effect estimate
In the a population with cases and non-cases, blood cobalt level and age in years show a slight association (not completely independent). Therefore, the case-only ICR must be multiplied by the S-E OR_c/nc_ to be ICR_c/nc_ according to Eq. (3).	Explanation
logit P(S = 1) = γ_0_ + γ_1_E ICR_CO_ = exp.(γ_1_)	Eq. (4) Eq. (5)
ICR_CO_: 1.14 (95% CI 1.03–1.37)	Effect estimate
When only the cases were analyzed (case-only approach), the case-only ICR was 1.14 (1.03–1.37), with a statistical significance (a positive interaction effect).	Explanation
${\mathrm{ICR}}_{\mathrm{c}/\mathrm{nc}}=\frac{{\mathrm{RR}}_{\mathrm{s}\mathrm{e}}}{{\mathrm{RR}}_{\mathrm{s}}{\mathrm{RR}}_{\mathrm{e}}}=\left(\frac{\mathrm{ag}}{\mathrm{c}\mathrm{e}}\right)\left(\frac{\left(\mathrm{c}+\mathrm{D}\right)\left(\mathrm{e}+\mathrm{F}\right)}{\left(\mathrm{a}+\mathrm{B}\right)\left(\mathrm{g}+\mathrm{H}\right)}\right)=\left({\mathrm{ICR}}_{\mathrm{CO}}\right)\left(\mathrm{S}-\mathrm{E}\ {\mathrm{OR}}_{\mathrm{c}/\mathrm{nc}}\right)$	Eq. (2)
ICR_CO_: 1.14 (1.03–1.37) × S-E OR_c/nc_: 1.06 (95% CI 1.03–1.10)	
ICR_c/nc_: 1.21 (95% CI 1.06–1.51)	Effect estimate
The ICR_CO_ multiplied by the S-E OR_c/nc_ produced the ICR_c/nc_ of 1.21 (95% CI 1.06–1.51).	Explanation

In this example, the environmental exposure (blood chromium level) and the susceptibility factor (blood glycohemoglobin level) are independent in the population with cases and non-cases. Therefore, the case-only ICR itself can be used as the ICR acquired from the population with cases and non-cases without a conversion. (This will be explained in the first discussion section in detail.) However, the ICR acquired from the population with cases, and non-cases was a statistically insignificant ICR because of a relatively wide confidence interval. This problem was solved by applying the case-only approach, producing a slightly decreased ICR with a statistical significance (a narrower confidence interval). A possible protective (negative) interaction effect between blood chromium levels and blood glycohemoglobin levels can be inferred from this example.

### The positive interaction effect between blood cobalt level and old age on albuminuria (micro and macro)

As the second example, Table 6 provides the sequential processes of applying the case-only approach in estimating the interaction effect between blood cobalt level and age in years on albuminuria. All these sequential processes follow the sequential processes provided in subsection 2.3: (i) Firstly, a 1 μg/L difference in blood cobalt level resulted in the fold-difference in the odds of albuminuria 1.09 (95% CI 0.98–1.20) times, without a statistical significance. (ii) Secondly, the 1-year difference in age resulted in the fold-difference in the odds of albuminuria by 1.05 (95% CI 1.04–1.05) times. (iii) Thirdly, when a 1 μg/L difference in blood cobalt level (mcg/L) and a 1-year difference in age coincide, the multiplicative ICR is 1.13 (95% CI 0.99–1.37), with statistical insignificance. (iv) Fourthly, in the population with cases and non-cases, blood cobalt level and age in years show a slight association, not completely independent (S-E OR_c/nc_: 1.06 (95% CI 1.03–1.10)). Therefore, the case-only ICR must be multiplied by the S-E OR_c/nc_ to be ICR_c/nc_ according to Eq. (2). (v) Finally, when only the cases are analyzed (case-only approach), the case-only ICR is 1.14 (1.03–1.37), with a statistical significance (a positive interaction effect). (vi) By multiplying S-E OR_c/nc_ by the ICR_CO_ calculated, the ICR_CO_-adjusted, 1.21 (95% CI 1.06–1.51), was produced.

In this example, the environmental exposure (blood cobalt level) and the susceptibility factor (age in years) are not independent in the population with cases and non-cases. Therefore, the case-only ICR must be multiplied by the S-E OR_c/nc_ to produce the ICR_c/nc_ according to Eq. (2). The ICR acquired from the population with cases, and non-cases showed a statistically equivocal ICR (1.13 (95% CI 0.99–1.37)). However, by applying the case-only approach, the ICR_CO_-adjusted showed a slightly higher ICR with a statistical significance (1.21 (95% CI 1.06–1.51). Therefore, a possible aggravating (positive) interaction effect between blood cobalt levels and ages in years can be inferred from this example.

## Discussion

Many previous studies dealt with various aspects of the case-only approach, usually in the context of gene-environment interaction studies or gene-gene interaction studies [5, 7, 9, 11, 14]. Some studies compared the case-only ICR with the ICR from the case-control design, whereas others compared the case-only ICR with the ICR from the population with cases and non-cases. This study incorporated all previous literature and systematically organized the provided logic and equations. From this effort, various definitions and equations for the ICR in the case-only design can be established compared to the ICR in the population with cases and non-cases (cohort/case-control studies). This systematic organization of concepts from 3 study designs is the original contribution of this study.

Furthermore, this study extended the case-only approach, which had been used usually in gene-environment interaction or gene-gene interaction studies, to a more general concept of the interaction effect estimation between susceptibility factors and environmental exposures. If the independence assumption between a susceptibility factor and an environmental exposure is fulfilled, even though the ‘gene’ is replaced with the ‘susceptibility factor,’ the same equations can be applied. Therefore, the case-only approach can also be applied to environmental epidemiology.

### The preventive (negative) interaction effect between blood chromium levels and glycohemoglobin levels on albuminuria (micro and macro)

The adverse effect of chromium on kidney function was reported in some previous literature [19, 20]. Glycohemoglobin level ≥ 6.5% is a diagnostic criterion for diabetes mellitus and is naturally associated with diabetic nephropathy [21]. Albuminuria, including micro-albuminuria and macro-albuminuria, has been used both as a useful initial marker for kidney damage and a marker associated with an increased risk of progressive renal diseases [22, 23]. However, a possible protective interaction effect is being increasingly reported for the interaction effect between chromium exposure and diabetic chronic kidney disease, based on improved glucose tolerance and insulin sensitivity [24–28].

The result of this study illustrates well a protective interaction effect between blood chromium level (environmental exposure) and blood glycohemoglobin level (susceptibility factor) on the albuminuria status (outcome). This protective interaction effect of chromium on diabetic patients with nephropathy can be used for establishing a future effective treatment strategy for diabetic nephropathy. For example, a study reports a possible positive effect of prescribing a nano chromium metal-organic framework on diabetic chronic kidney disease patients [24].

### The aggravating (positive) interaction effect between blood cobalt levels and old ages on albuminuria (micro and macro)

The effect of blood cobalt levels on kidney function is not yet established, with only a few studies reporting possible adverse effects, mainly in experimental animals [29]. However, the effect of aging on decreasing kidney function is relatively well established [30, 31]. Furthermore, the fact that this aging kidney is susceptible to various toxic substances is well known through numerous studies [32–35]. From these pieces of evidence, we can infer that the aging kidney could be more susceptible to the possible toxic effect of cobalt, even if it is almost non-toxic to the young kidney.

The result of this study illustrates well this toxin-susceptible feature of the aging kidney (susceptibility factor) to cobalt exposure (environmental exposure). As a marker of kidney damage, the proportion of albuminuria was greater in the older subjects. The result of this study can be used to devise a protective environmental health strategy for aging people with an increased possibility of exposure to heavy metals, such as cobalt.

## Conclusion

This study summarized the previously reported logic and equations about the case-only approach systematically. In particular, the associated definitions and equations are collectively summarized from the cohort and case-control (study designs with cases and non-cases) to case-only studies. By substituting the ‘susceptibility factor’ concept from environmental epidemiology for the conventional ‘gene’ concept from genetic epidemiology, this study broadened the applicability of the case-only approach to broad environmental health topics. If the independence assumption between a susceptibility factor and an environmental exposure in the population with cases and non-cases is kept, this case-only approach can provide a more precise interaction effect estimate than that from study designs with cases and non-cases (cohort/case-control studies). Finally, 2 analysis examples of the case-only approach using the US NHANES datasets were explained. The protective interaction effect between blood chromium levels and blood glycohemoglobin levels and the aggravating interaction effect between blood cobalt levels and increasing ages on the incidence of albuminuria must be investigated meticulously in future studies. In summary, the case-only approach can be a useful approach not only in genetic epidemiology but also in environmental epidemiology.

## Supplementary Information

**Additional file 1: Supplementary material A.** The used R codes for the statistical analyses. **Supplementary material B.** The S-E independence in the controls cannot replace the S-E independence in the population with cases and non-cases [1]. **Supplementary material C.** How strong a rare disease assumption is required for the equality between S-E OR_c/nc_ and S-E OR_control_ [1]. **Supplementary material D.** Violation of independence: confounder [1].

## Data Availability

All data used in this article are available on the National Health and Nutrition Examination Survey homepage (https://wwwn.cdc.gov/nchs/nhanes/).
