# Supplementary material for: Case-only approach applied in environmental epidemiology: 2 examples of interaction effect using the US National Health and Nutrition Examination Survey (NHANES) datasets
Source: BMC Med Res Methodol. 2022 Sep 29;22:254. doi: 10.1186/s12874-022-01706-6 (PMC9520813; doi:10.1186/s12874-022-01706-6)
Supplement: Supplementary file 1 — Additional file 1: Supplementary material A. The used R codes for the statistical analyses. Supplementary material B. The S-E independence in the controls cannot replace the S-E independence in the population with cases and non-cases [1]. Supplementary material C. How strong a rare disease assumption is required for the equality between S-E ORc/nc and S-E ORcontrol [1]. Supplementary material D. Violation of independence: confounder [1]. [file 12874_2022_1706_MOESM1_ESM.docx]

**SUPPLEMENTARY MATERIALS**

**Supplementary material A. The used R codes for the statistical analyses**

**A-1. For the negative interaction effect between blood chromium level and glycohemoglobin level on micro- and macro-albuminuria**

> model=glm(URDACT2~LBXBCR,family=binomial,data=data)

> model=glm(URDACT2~LBXGH,family=binomial,data=data)

> model=glm(URDACT2~LBXBCR2*LBXGH,family=binomial,data=data)

> model=glm(LBXBCR2~LBXGH,family=binomial,data=data)

> model=glm(LBXBCR2~LBXGH,family=binomial,data=data2)

**A-2. For the positive interaction effect between blood cobalt level and old age on micro- and macro-albuminuria**

> model=glm(URDACT2~LBXBCO,family=binomial,data=data)

> model=glm(URDACT2~RIDAGEYR,family=binomial,data=data)

> model=glm(URDACT2~LBXBCO2*RIDAGEYR,family=binomial,data=data)

> model=glm(LBXBCO2~RIDAGEYR,family=binomial,data=data)

> model=glm(LBXBCO2~RIDAGEYR,family=binomial,data=data2)

**Supplementary material B. The S-E independence in the controls cannot replace the S-E independence in the population with cases and non-cases [1]**

| \|  \| S+ \| S- \| \| --- \| --- \| --- \| \| E+ \| g+H  (120) \| e+F  (5,880) \| \| E- \| c+D  (280) \| a+B  (13,720) \| | |
| --- | --- | --- | --- | --- | --- | --- | --- | --- | --- | --- |
| S-E association in a cohort study (D+ and D-)  S-E OR_c/nc_=1.0  ICR_c/nc_=2.5 | |
| \|  \| S+ \| S- \| \| --- \| --- \| --- \| \| E+ \| g  (48) \| e  (470) \| \| E- \| c  (22) \| a  (549) \| | \|  \| S+ \| S- \| \| --- \| --- \| --- \| \| E+ \| H  (72) \| F  (5,410) \| \| E- \| D  (258) \| B  (13,171) \| |
| S-E association in the case-only study (D+)  ICR_CO_=2.5 | S-E association in the controls (D-)  S-E OR_control_=0.7 |
| The ICR_co_ is equal to the ICR_c/nc_ since the S-E OR_c/nc_ is 1.0.  The S-E OR_control_ of 0.7 is a poor proxy for the S-E OR_c/nc_ of 1.0. | |
| \|  \| S+ \| S- \| \| --- \| --- \| --- \| \| E+ \| g+H  (452) \| e+F  (5,548) \| \| E- \| c+D  (548) \| a+B  (13,452) \| | |
| S-E association in a cohort study (D+ and D-)  S-E OR_c/nc_=2.0  ICR_c/nc_=1.0 | |
| \|  \| S+ \| S- \| \| --- \| --- \| --- \| \| E+ \| g  (293) \| e  (599) \| \| E- \| c  (132) \| a  (538) \| | \|  \| S+ \| S- \| \| --- \| --- \| --- \| \| E+ \| H  (159) \| F  (4,949) \| \| E- \| D  (416) \| B  (12,914) \| |
| S-E association in the case-only study (D+)  ICR_CO_=2.0 | S-E association in the controls (D-)  S-E OR_control_=1.0 |
| The ICR_co_ is not equal to the ICR_c/nc_ since the S-E OR_c/nc_ is 2.0.  The S-E OR_control_ of 1.0 is a poor proxy for the S-E OR_c/nc_ of 2.0. | |

S: Susceptibility factor. E: Environmental exposure.

**Supplementary material C. How strong a rare disease assumption is required for the equality between S-E OR_c/nc_ and S-E OR_control_ [1]**


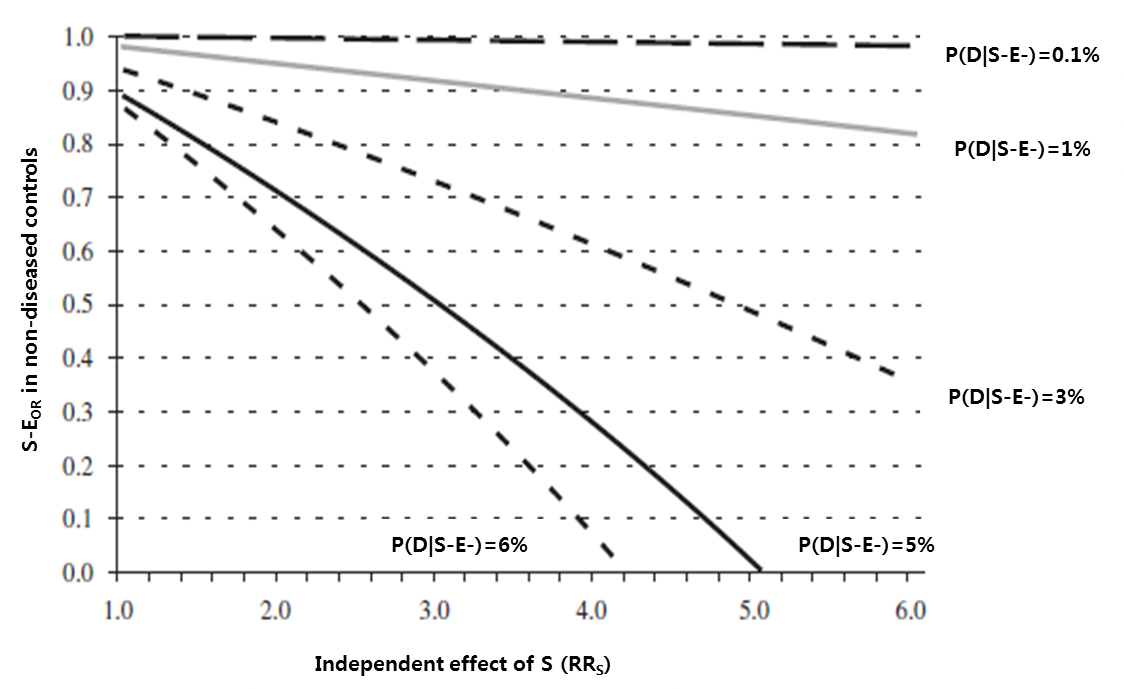


**Supplementary material D. Violation of independence: confounder [1]**


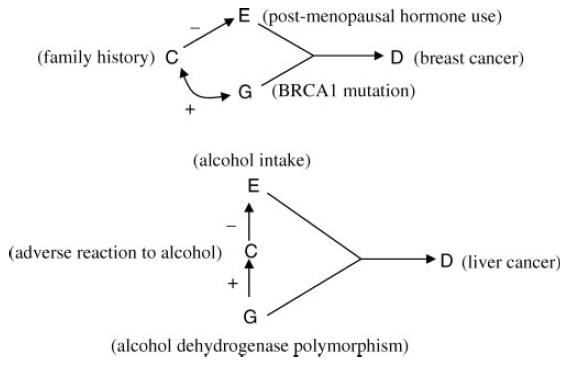


**REFERENCES**

1. Gatto NM, Campbell UB, Rundle AG, Ahsan H: **Further development of the case-only design for assessing gene-environment interaction: evaluation of and adjustment for bias**. *International journal of epidemiology* 2004, **33**(5):1014-1024.
